# ”What’s up with price controls?” Stakeholders’ views on the regulation of pharmaceutical pricing in Malaysia

**DOI:** 10.1371/journal.pone.0291031

**Published:** 2023-12-07

**Authors:** Ye Shing Lourdes Loh, Audrey K. L. Siah, Sharon G. M. Koh, Wing Loong Cheong, Tin Tin Su

**Affiliations:** 1 Department of Economics, School of Business, Monash University Malaysia, Bandar Sunway, Malaysia; 2 School of Pharmacy, Monash University Malaysia, Bandar Sunway, Malaysia; 3 Jeffrey Cheah School of Medicine and Health Sciences (JCSMHS), Monash University Malaysia, Bandar Sunway, Malaysia; Lahore Medical and Dental College, PAKISTAN

## Abstract

Escalating costs have made the accessibility of drugs one of the biggest challenges faced by the Malaysian government. The government agreed to regulate drug prices by means of external reference pricing, but its proposed policy had a setback owing to much opposition from the pharmaceutical industry. The policy did gain support from the public and from non-governmental organisations because it ensured easy access to affordable medicines. Comments from public consultations with key stakeholders were used to explore stakeholders’ perceptions of the external reference pricing policy. A total of 140 comments were analysed for this study. Stakeholders’ views were classified as being from the Socioeconomic, industrial, and government sectors. To summarise, the government must carefully manage and consider stakeholders’ views to ensure a sound policy. Using Mendelow’s stakeholder mapping, this study mapped out stakeholders’ views in a systematic approach. The classification of different stakeholders’ views and recommendations led to suggestions for reviewing current practices in pharmaceutical pricing regulations in the Malaysian healthcare system. The analyses can be extended to other countries that face similar concerns.

## Introduction

As concerns about global health equity increase over time, policymakers have advocated more and more strongly for pharmaceutical pricing regulations to ensure that healthcare is accessible for all. The escalating costs of drugs have become one of the biggest challenges in this arena. National governments must try to safeguard the fundamental rights of patients so they can have access to treatment, especially in developing countries that have to deal with the pressures of increasing costs of healthcare and medicines [1]. The high costs could make medicines less affordable for patients and increase the government’s burden of spending for healthcare. Healthcare expenditures account for about 60% of the total expenditures in developing countries [1]. Over the past few decades, there has been continuous double-digit growth in the medical inflation rate in Malaysia [2], and this has been an obstacle to achieving United Nations Sustainable Development Goal Three, which is having affordable and accessible healthcare. Because there is a lack of pricing controls in Malaysia, increasing prices could result in larger out-of-pocket payments for medicines, especially when open market forces are a major determinant of these prices [3]. Studies have shown that the free market system has led to an overall increase in the use of generic medicines, with a 31% to 402% markup on their final prices [3]. Wong et al. showed that the Malaysian procurement prices in the public sector were 1.5 times the international reference prices (IRPs) and in the private sector were 8.4 times the IRPs [4]. In the same study, Wong et al. also observed that there were large variations in the prices of medicines in the private health sector and that paying high prices could potentially worsen a patient’s welfare [4]. You et al. found that medications that cost more than the daily wage of the lowest paid unskilled government worker were considered unaffordable for local patients [5]. Taking non-communicable diseases as an example, some originator medicines for gastrointestinal diseases cost 6.4 to 8.1 days’ wages for a minimum wage worker in Malaysia [6]. As a result, high medical expenses could burden low-income patients, particularly patients with chronic conditions such as cancer [7]. The excessive financial burden could worsen health conditions because patients might switch to less expensive medications or even delay or forgo a recommended treatment [7]. This could not only hinder patients’ physical health but also impact their overall welfare.

In an attempt to monitor prices in the private healthcare market, the Malaysian government introduced the Medicine Price Unit (MPU), a subdivision of the Pharmaceutical Service Division (PSD), in 2005. The MPU created a database that serves as a public reference for medicine pricing, and it can be useful in enhancing the transparency of pricing [8]. However, the database was deemed not effective due to a lack of enforcement of regulations. Most healthcare providers rely on free market forces to determine medicine prices [9]. In the current setting of the Malaysian healthcare ecosystem, it can be assumed that, if the pricing controls for the pharmaceutical market are being implemented, such regulations would be helpful in controlling the prices of medicines, as well as improving the affordability of essential medicines.

Given the imperfections of the healthcare market, pharmaceutical pricing regulations are often seen as a remedy for the escalating prices of medicines. To assess the increase, former Malaysian Health Minister Dzulkefy Ahmad first announced the consideration of pharmaceutical pricing regulations by adopting external reference pricing (ERP) in May 2019 [10]. ERP compares the prices of pharmaceutical products in several countries in order to set benchmark prices. This system has been successfully adopted by many European countries as a price control measure [11]. The “reference countries” for the ERP policy should fulfill five criteria of the World Health Organisation’s (WHO’s) Pharmaceutical Pricing Policies. According to the WHO guideline [12], the five criteria are listed as follows:

1. Geographical proximity
2. Income level (refers to comparable gross domestic product [GDP] levels)
3. Availability of medicines
4. Country of origin (similar socioeconomic conditions)
5. Market size

For countries that have not implemented any form of pharmaceutical pricing regulations, such as Malaysia, the ERP policy could be considered a straightforward and simple approach [13]. It could fufill the cost control requirement of the government to ensure that domestic medication prices were fair [14]. Therefore, this study focused on examining the perceptions of affected stakeholders of the implementation of the ERP policy.

To evaluate the potential impact of the ERP policy, the Malaysian government relied on the WHO standard to identify 20 reference countries. The full list of countries has not been disclosed by the government [15]. To understand the feasibility of the ERP policy, two analyses have been conducted by the government:*Cost Benefit Analysis of The Medicines Price Mechanism* (CBA 1.0) and the *Comprehensive Cost-Benefit Assessment on the Medicine Pricing Policy* (CBA 2.0), published in December of 2021. The ERP policy might adversely impact the healthcare sector with a loss of up to RM4.30 for every RM1 saved or a loss of up to RM31 billion in wages for the next 15 years [16]. Furthermore, price regulations could result in adverse effects if the proposed policy were not evaluated carefully. Stakeholders have raised concerns about the findings of CBA 1.0 and CBA 2.0 during the public consultation period, which will be further elaborated in the results section of this study. The study has identified the following gaps in CBA 1.0 and CBA 2.0.

### Lack of stakeholder inclusiveness

Pharmaceutical pricing regulation has received mixed reactions from the pharmaceutical industry and healthcare service providers, and the pharmaceutical industry has become the greatest opponent to implementing ERP [17]. Furthermore, some academicians have argued that governments should not interfere with the private sector [18].

Additionally, non-governmental organisations (NGOs) have expressed concerns that the reliability of the findings of CBA 1.0 and CBA 2.0 may be questionable due to the lack of stakeholder inclusiveness [19, 20]. Lim posited that the stakeholders involved were mostly industry players (i.e., private hospitals) and pharmaceutical companies [20]. This has remained one of the biggest challenges for national governments trying to implement an effective healthcare policy because these stakeholders could choose to prioritise their self-interests when implementing the policy. Hence, stakeholder inclusiveness is particularly important for ensuring that the policy is effective. We must ensure that all key actors’ concerns have been identified and taken into consideration. This is crucial for both the government and the affected stakeholders, as they try to achieve a consensus about a policy.

### Including stakeholders’ dynamics in policymaking

Like other policies, pharmaceutical pricing regulations are heavily influenced by stakeholders’ interests and power. One of the policymakers’ concerns is assessing and influencing stakeholder dynamics [21]. Oxman et al. explained that stakeholder dynamics could be the power relations among stakeholders and the interests of various stakeholder groups, which are crucial factors in the policymaking process [21]. Exploring stakeholder dynamics allows the researcher to acknowledge the “multiple and independent interactions between stakeholders” [22]. Understanding stakeholder dynamics could help in evaluating stakeholders’ levels of influence on the development of strategy (i.e., public policy) [23]. It is useful for policymakers to have a clear idea of which stakeholder to engage with to ensure successful policy implementation. However, the stakeholder dynamics in the current context have not yet been elucidated. This work addresses the underlying stakeholder dynamics in pharmaceutical pricing regulation policymaking.

Hence, this study contributes to the limited literature on stakeholders’ underlying views on implementing pharmaceutical pricing regulations. The study also provides recommendations from key stakeholders on the current healthcare policy. The research is designed to provide new evidence for identifying stakeholders’ influences, including their level of power and interests, as a critical component of influencing the ERP policy. Fundamentally, this research aimed to capture the views on and obstacles to pharmaceutical pricing regulation and provide insights that could improve Malaysia’s healthcare system.

The study aimed to answer the research questions (RQs) as follows:

1. What are the perceptions of various stakeholders about implementing the ERP policy?
2. What are the main concerns of various stakeholders in regard to implementing the ERP policy?
3. What are stakeholders’ recommendations for the current Malaysian healthcare policy?

## Data and methods

### Design and setting

This study aimed to identify the potential influences of relevant stakeholders and the stakeholders’ primary considerations in pharmaceutical pricing regulations. To explain the research questions, a qualitative approach was adopted, which could be the best fit for the study’s exploratory nature. Using a qualitative approach allowed us to discover the reasons behind the observed patterns [24], and it was particularly useful for enabling a deeper understanding of personal views on the implementation of pharmaceutical pricing regulation [25]. This study used a multiple-method approach across two phases. It collected online comments from the Malaysian government’s Unified Public Consultations (UPC) website and also obtained statements released by relevant stakeholders. The UPC website is a government portal that acts as a platform on which Malaysians can post their views on how public policies and regulations are designed and enforced. For the context of implementing the ERP policy, the government collected comments from relevant stakeholders, such as healthcare providers and patient groups. The comments were evaluated by qualitative content analysis. The detailed data collection process is described in the next section.

### Data collection

### Phase 1: Understanding the context

In the first phase, the main task was to gain a preliminary understanding of the medicine price control policy that the Malaysian government proposed in 2019. This phase involved mapping out which stakeholders were more likely to be involved in the policy-making process and potentially influence decisions about implementing the medicine price control policy. This stage reviewed the extant literature to gain a deeper understanding of the Malaysian healthcare ecosystem and identify the key stakeholders of the healthcare system [6, 8, 13, 15, 16]. Further, the study assessed the closure reports of both CBA 1.0 and CBA 2.0, which listed stakeholders involved in the policymaking process and the number of meetings held with key stakeholders as the reference of stakeholder mapping.

### Phase 2: Qualitative content analysis of UPC comments

The second phase gathered public comments and official statements from the UPC website and two public consultations conducted between 5 January 2021 and 25 January 2021, and between 29 November 2021 and 6 December 2021. Both public consultations involved key relevant stakeholders in relation to CBA 1.0 and CBA 2.0. We only included comments related to the implementation of the ERP policy (i.e., they were the inclusion criteria). There were 53 comments from the first public consultation and 87 from the second, totalling 140 comments after all unrelated and repeated comments from the original total of 187 comments were removed from the UPC portal. The statements are publicly available on the public consultation website. This allowed us to discover the stances and recommendations of the commenters on implementing pharmaceutical pricing regulation in Malaysia.

According to Schreier, content analysis is used to classify written and oral statements with similar meanings [26]. This approach could be beneficial for making inferences about the meaning of written texts, because it systematically conceptualising the meaning of a text that is partly obscured [27]. Qualitative content analysis could be used as a research method as it “provides profound insight into a situation which is not limited by existing viewpoints or methodologies.” [28]. Because online portals have become the most common way of expressing opinions and generating conversations, social researchers often using content analysis to analyse social media posts and comments [29, 30].

The sample demographics were not fully identified because some comments, particularly those in the second consultation, remained anonymous on the public consultation website. However, through the public consultation webpage, we were able to derive some brief information about the stakeholders involved in the public consultation, as shown in Table 1. The affected stakeholders of the ERP policy, included patient groups, insurance providers, pharmaceutical companies, and healthcare providers, and they were invited to provide their comments on the UPC portal. In addition, some comments made by prominent stakeholders and healthcare professionals were visible during the first public consultation. All the comments were made publicly available, and no conflict of interest was identified.

**Table 1:** Overview of data collected from both public consultations.

	First Consultation (UPC 1.0)	Second Consultation (UPC 2.0)
**Number of Comments**	53	87
**Consultation Period**	5 January 2021 to 25 January 2021	29 November 2021 to 6 December 2021
**Identity of Stakeholders**	Patient groups, Insurance providers’, Pharmaceutical Companies, Healthcare Providers, Government Agencies	Patient groups, Pharmaceutical Companies, Private Hospitals/Clinics, Pharmacies and Distributors, Insurers, Government agencies, Businesses in Medical Tourism, Investors.
**Referred Analysis**	CBA 1.0	CBA 2.0

### Data analysis

All comments obtained from the UPC website followed a content analysis approach adopted by Brown et al. and Cho and Lee [29, 31]. The coding process focused on understanding commenters’ thoughts about three different aspects of the policy: (1) the responses of Malaysians about the implementation of medicine price controls; (2) their comments about the design and findings of CBA1.0 and CBA2.0; and (3) their recommendations on what should be done with the current Malaysian healthcare policy.

By obtaining a sufficient understanding of the topic by reading through relevant news and comments,a total of 140 comments from the portal were organised into two documents (see Table 1 for the overview). Open coding using Nvivo V.12 identified key themes from the comments. The codes were identified from the UPC comments and refined through a thematic analysis approach, in which the research team worked to develop a list of codes. After identifying a series of codes, the researchers sought connections between the themes identified and clustered those with similar meanings. Lastly, the final themes for the study were presented in Table 2. The analysis was done following an established practice by Saldana [32] for thematic analysis.

**Table 2:** Abbreviations used in quoted UPC comments.

Acronym	Complete Term
APHM	Association Of Private Hospitals, Malaysia
DCA	Drug Control Authority
GP	General Practitioner
MCPG	Malaysian Community Pharmacy Guild
MITI	Ministry of Investment, Trade and Industry
MMA	Malaysian Medical Association
MNC	Multinational companies
MOH	Ministry of Health
MOPI	Malaysian Organisation of Pharmaceutical Industries
MPC	Malaysia Productivity Corporation
PhAMA	Pharmaceutical Association of Malaysia
PhRMA	Pharmaceutical Research and Manufacturers of America
STEM	Science, technology, engineering and mathematics

Using the refined version of Mendelow’s mapping technique adapted from research studies in the healthcare sector [33, 34] as well as the work of Scholes and Johnson [23], the results were obtained from the specific questions for determining the power and interest level of the involved stakeholders. Mendelow’s stakeholder mapping is a commonly used method that allows the researcher to understand the impact of and concerns about the policy at different stages [35, 36]. By mapping out the power and positions of key stakeholders, we can assess the likelihood of the implications and the acceptability of new policies. [36]. Results from the thematic analysis were classified as socioeconomic, industrial, or governmental to reflect the different perspectives on the pharmaceutical pricing policy from different stakeholders.

## Results

### Mendelow’s stakeholder mapping

**Fig 1:**
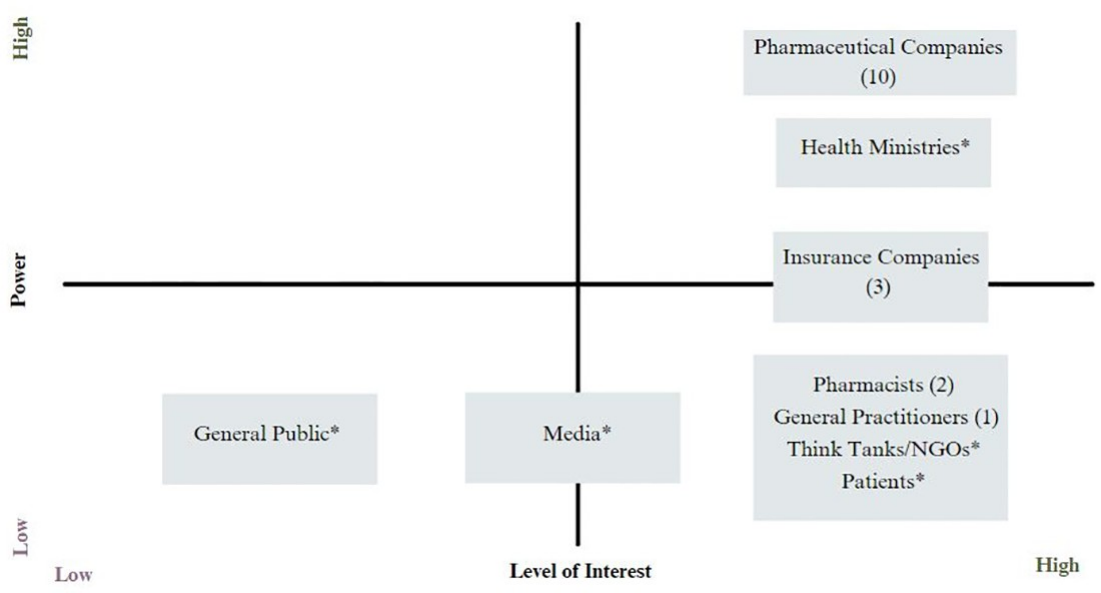
Mendelow’s stakeholder matrix showing stakeholders’ levels of power and interest. *The number of meetings could not be determined; however, the stakeholder has been involved in the policy making process with certain degrees of influence.

The initial list of stakeholders was drawn up after reviewing the relevant literature (Fig 1). The decision criteria for the power level and interest level are listed below:

**Determinants of power level:**

1. Possession of resources
2. Dictation of alternatives
3. Authority
4. Level of influence

**Determinants of interest level:**

1. How much awareness do stakeholders have of the impact?
2. How interested is the stakeholder group in the policy (i.e., what are their expectations for the policy)?

The stakeholders’ position was determined based on the key factors outlined below: 1. The official records of meetings held between governmental leaders and key stakeholders (the numbers in the brackets indicate the total number of meetings held between January 2019 and October 2020 as this reflects the authority to influence the policymaking process). 2. Awareness of each stakeholder about the proposed ERP policy was considered the stakeholder’s interest level. This is determined by the public statements made by the stakeholders. After determining who the key stakeholders were, the study analysed the stakeholders’ viewpoints on the ERP policy.

As shown in Fig 1, the stakeholder analysis revealed that pharmaceutical companies and health ministries had the highest power and interest levels in terms of the ERP policy. The general public demonstrated low power and interest, as the involvement of the public in government meetings was minimal for pricing regulations, and it was unlikely that comments from the general public would be found on the UPC platform. Furthermore, the media were portrayed as having low power and medium interest; there were only a few media outlets, such as Malaysiakini, CodeBlue, The Malaysian Insight, and The Malay Mail, that covered news relevant to pricing regulations. Stakeholders who demonstrated low power but high interest were patients, NGOs, and general practitioners. Although two patient advocacy groups had been consulted in CBA 2.0, their small number was not representative enough of their stakeholder category to be significant (see S1 Appendix for a full analysis).

### Public consultation

A total of 140 comments were analysed. The public consultation identified the perceptions of key stakeholders on implementing the ERP as a price control mechanism. Various stakeholders addressed the main challenges of implementing pharmaceutical pricing regulation as well as the limitations of the existing healthcare system in Malaysia. All comments were analysed using thematic analysis, where all themes were grouped into three different levels (Table 3). A full list of the abbreviations used in the comments by our key informants is available in Table 2.

**Table 3:** Summary of results.

Stakeholder Level	Key Themes	Findings	Key Informant Quote
Socio-economic	Economic impact of the policy	Implementing ERP could impact the economic development of the healthcare market.	*“Pharmaceutical manufacturers, especially innovative companies, will potentially defer the launch of new innovative medicines due to the unattractive market landscape with such a price control.” (UPC 2.0)*
	Welfare impact of the policy	Price controls could determine the accessibility of essential medicines for patients and the general public.	*“I would think that medicine price control is essential. The high cost of innovative drugs will deny a patient’s access to affordable, innovative medicine. As a result, it will lead to financial catastrophe and impoverishment for patients who pay for their own medicine (long term) out-of-pocket.” (UPC 2.0)*
Government	Policy (re)evaluation	Policy evaluation is needed as there are unsolved issues in the analysis done previously by the government, where the data source of the analysis still needs to be clarified for the CBA	*“Need for a full study report with details and information: The preliminary findings are just results presented in numbers, without clear and specific methodology, assumptions, supporting data and calculation process for the public to validate and review each of the findings.” (UPC 2.0)*
	Healthcare reform	There is a need for healthcare reform before considering implementing pharmaceutical pricing regulations.	*“MCPG would agree to MOH’s proposal on medicine price control ONLY if universal health coverage and dispensing separation are in place.” (UPC 1.0)*
Industry	Prospects in the pharmaceutical Industry	Pharmaceutical pricing regulations might affect the overall prospects in the pharmaceutical industry, such as affecting R&D investment, sustainability, fair competition in the industry, and reduced profitability.	*“We are already facing difficult time dealing with ever increasing costs of complying and updating with revolving regulatory demands, raw and packaging material, logistics, operations and finally cut-throat competition among locals and imported drugs suppliers. Thus, the profit margin is getting very thin.” (UPC 2.0)*

### Industry-level factors

#### Prospects in the pharmaceutical industry

In the context of industry-level factors, in order for healthcare providers to keep up with regulatory demands, the increasing production costs of the production line, as well as cutthroat competition in the industry, have caused local pharmaceutical companies to operate on a thin profit margin. The implementation of price controls would further worsen the situation.

*“We are already facing [a] difficult time dealing with ever-increasing costs of complying [with] and updating revolving regulatory demands, raw and packaging material, logistics, operations and finally cut-throat competition among locals and imported [drugs] suppliers. Thus, the profit margin is getting very thin.”*

*(UPC 2.0)*

*“Pharmaceutical companies are significant investors in Malaysia, not just through creating jobs, but also through education and clinical trial activity. These activities will be negatively impacted if there is a significant reduction in profit margins brought in by medicine price controls.”*

*(UPC 2.0)*

The comments also raised concerns about the economic sustainability of pharmaceutical companies and private healthcare providers. The decline in profits might result in reduced incentives for healthcare providers to stay in the healthcare market.

*“Government healthcare is sustainable because it is paid by the government without a price tag for the patients. If private healthcare particularly the GPs and some private hospitals, cannot stay afloat, they will be forced to close down.”*

Hence, key informants from the healthcare industry had similar views on remaining in the free market and how it could be the best practice for the current healthcare market, as the operating costs could vary depending on location, fixed and variable costs.

*“Medicine price[s] in the private market should be based in free market principles [because] the cost[s] of operations for the retail pharmacies, private hospitals and clinics vary depending on their location, fixed and variable costs.”*

*(UPC 2.0)*

Furthermore, key informants believed that ERP would curtail the incentive to invest in the research and development (R&D) of new drugs.

*“Access to medicines and clinical investments will be reduced if the country [loses] attractiveness for having a sustainable environment for investments.”*

*(UPC 1.0)*

### Socio-economic level factor

#### The economic impact of the policy

One of the main concerns of the commenters was that implementing the ERP could impact the economic development of the healthcare market. Most thought that price controls would reduce the competitiveness of the local healthcare market.

*“Pharmaceutical manufacturers especially the innovator companies will potentially defer [the] launch of new innovative medicines due to unattractive market landscape with such price control[s].”*

*(UPC 2.0)*

#### Welfare impact of the policy

Key informants posting on the UPC platform provided their views on the potential impact of the ERP on the welfare of the general public. They commented that price controls might affect the accessibility of healthcare products or services because the number of pharmaceuticals available in the market might be reduced.

*“By controlling the medicine price, the MNC pharma companies will delay launching the innovative medicines. Rakyat (people) will suffer a poorer prognosis due to [the inaccessibility of] the latest breakthrough medicine.”*

*(UPC 2.0)*

In the context of the industrial-related factors, key informants contended that reduced profitability in business might result in the cessation of business. Furthermore, business closures reduce employment opportunities in the healthcare sector and can contribute to the downsizing of businesses and, therefore, unemployment.

*“If the proposed policy is implemented [,] pharmaceutical companies will not be able to consider further investment[s] in Malaysia, and this will result in a reduction in clinical trials and loss of STEM jobs.”*

*(UPC 1.0)*

*“As a result, jobs will be reduced, [and] many employees will suffer from unemployment.”*

*(UPC 2.0)*

On the other hand, key informants, mainly the patient group, showed their support for implementing the ERP. Highly specialised drugs are often expensive, and the cost may place a financial burden on patients that could result in their opting out of treatment.

*“I would think that [a] medicine price control is essential. High-cost innovative drug[s] will deny a patient access to affordable innovative medicine. As a result, it will lead to financial catastrophe and impoverishment for [the] patient who pays for their own medicine (long term) out-of-pocket.” (UPC 2.0)*

### Government (Public policy) level factor

#### Policy (re)evaluation

Most key informants highlighted a need to re-evaluate the suitability of imposing pharmaceutical pricing regulations in the Malaysian context. Some key informants pointed out that were unsolved issues in the previous analyses of CBA1.0 and CBA 2.0 done by the government, where the data source, methodology and calculation process of the analysis still needed to be clarified.

*“[There is a] need for a full study report with details and information: The preliminary findings are just results presented in numbers, without [a] clear and specific methodology, assumptions, supporting data and calculation process [that would allow] the public to validate and review each of the findings.”*

*(UPC 2.0)*

In addition, a conflict of interest remains one of the unresolved issues that might affect the final results of the CBA process.

*“We are particularly concerned over the serious conflict of interest in this collaboration project between [the] public sector (MITI, MPC) and the private sector (PhAMA, APHM, MOPI, MMA, PhRMA) [,] especially because the CBA 2.0 is funded by industry, and some of its members even sit on oversight committees.”*

*(UPC 2.0)*

Other than methodological issues in the CBA process, some informants mentioned that policies could be refined by considering the holistic view of diverse stakeholders that truly reflects the current best practice.

*“As such, we would like to stress that price control can only be done with proper implementation, [and] thorough consideration of all stakeholder interest and not erratic changes in pricing.”*

*(UPC 1.0)*

#### Healthcare reform

Key informants from the public consultation raised several issues related to the current healthcare system. They highlighted the necessity to review the existing national patent system to encourage the development of generic medicines in Malaysia.

*“In our opinion, even more savings can be achieved, even with only [one] product, with proper patent reviews and fast registration of generics to the market.”*

*(UPC 1.0)*

Besides reviewing the current patent system, rapid approval of generics could enhance market competition, which could reduce the prices of medicine through the free market mechanism.

*“The rapid approval [by the DCA] of biosimilars and generics once patents have expired will facilitate competition which will drive down prices.”*

*(UPC 1.0)*

Key informants divulged that universal healthcare should be prioritised over pharmaceutical pricing regulation.

*“MCPG would agree to MOHs proposal on medicine price control ONLY if universal health coverage and dispensing separation are in place.”*

*(UPC 1.0)*

Key informants from the pharmaceutical industry opined about the flexibility of pricing of pharmaceutical products, which could be the best practice for encouraging competition in the industry.

*“Hence if [the] pharma industry does not get flexibility of pricing, [it] will be hesitant to make further investments. Besides, a free pricing policy encourages competition and gives flexibility to customer[s] to negotiate, which is a healthy practice for [the] corporate [sector] as well as customers.”*

*(UPC 1.0)*

Besides price flexibility, some informants asserted that price transparency was important for ensuring fairer and more reasonable prices.

*“Private providers admit to includ[ing] hidden charges of their operating cost[s] in medicine prices. And suppliers apply different price scheme[s] when selling to their buyers. So we want prices of medicines that are reasonable and also transparent across all health facilities.”*

*(UPC 2.0)*

Notably, the pharmacist community proposed dispensing separation that sets a clear boundary between prescribing (by general practitioners) and dispensing (by pharmacists). This could be an effective method of controlling medicine prices where pharmacists have dispensing rights.

*“Dispensing separation or mandatory prescriptions issued by doctors must be in place to ensure [that] members of the public have a choice [as to] where to buy their medicines. Cost saving must be done with policy amendments to avoid [having] independent pharmacists [becoming] the victim of predatory tier pricing mechanisms.”*

*(UPC 2.0)*

In the public consultation, key informants provided recommendations on what aspects should be prioritised in the Malaysian healthcare system. These recommendations included reviewing the current patent system, enhancing the approval of generic medications, proposing universal healthcare coverage, and enhancing price flexibility and transparency, as well as separating the dispensing rights.

## Discussion

This study analysed the perceptions of key stakeholders on implementing the ERP through thematic analysis, guided by Mendelow’s stakeholder mapping to understand various key stakeholders’ levels of influence in the policy-making process. The study analysed key stakeholders’ underlying views of the feasibility of implementing pharmaceutical pricing regulation. This study differed from that of Ashraf and Ong [13] on stakeholder analysis, which synthesised key stakeholders’ positions and roles as well as their perceptions based on information collected from newspaper articles, speeches, conference presentations, and social media sites. This study, however, directly explored key stakeholders’ perceptions by analysing online comments, which offered qualitative evidence of the suitability of adopting pharmaceutical pricing regulations. The research findings contribute to the literature by increasing the understanding of stakeholder’s views on implementing the ERP and the obstacles inherent in the existing healthcare policy. This study also provided a more comprehensive view for policymakers that linked industry players, the general public, disadvantaged groups, and other agencies, which has been evident in the framework of successful stakeholder partnerships mentioned in Riege and Lindsay [37].

Stakeholder inclusion could be one of the important criteria for ensuring the efficacy of healthcare policy. Adams and Hess affirmed that the knowledge exchange between society and government is a major determinant of the legitimacy of public policy [38]. They specified that the CBA process and public consultation in Malaysia were critically inadequate to involve certain key and important stakeholders such as patient groups, consistent with the findings of Ashraf and Ong [13]. As suggested by Ashraf and Ong, a more holistic policy should consider the opinions of all stakeholders, whether they are positively or negatively affected by such a policy [13]. In this research, the findings have shown the underlying views of stakeholders from different levels through comments from the UPC, which demonstrated the challenges and concerns faced by different stakeholders. The study revealed that findings aiming to address RQ1 and RQ2 are often inextricably intertwined, as stakeholders would raise concerns about ERP implementation in the same breath as offering their perceptions. The findings have demonstrated mixed reactions, especially related to socioeconomic factors; some stakeholders were concerned about the accessibility of medicines as pricing regulations may have delayed the launch of innovative drugs. Kanavos et al. reviewed the effects of pricing regulations, particularly in the ERP policy about the availability of medicines across 54 studies. The results indicated that the ERP policy could have promising results in lowering pharmaceutical prices, but that delays in launching new drugs and reducing the availability of medications could be prevalent if the policy were not properly designed [39]. Similarly, this study has shown that the concerns about delays in drug launches are also non-negligible issues for developing countries like Malaysia. Patient groups argued that the high medication fees for treatment might lead to financial catastrophes for patient or their families. This was evident in a study conducted by the Asean CosTs In ONcology (ACTION) study group in 2015, which found that more than 75% of patients had experienced financial catastrophe or had died within a year of their diagnosis [40]. Recall what has been discussed earlier in this paragraph; the results have suggested that policymakers listen and carefully evaluate the potential impact of the policy and each stakeholder’s views before any decision is made.

**Fig 2:**
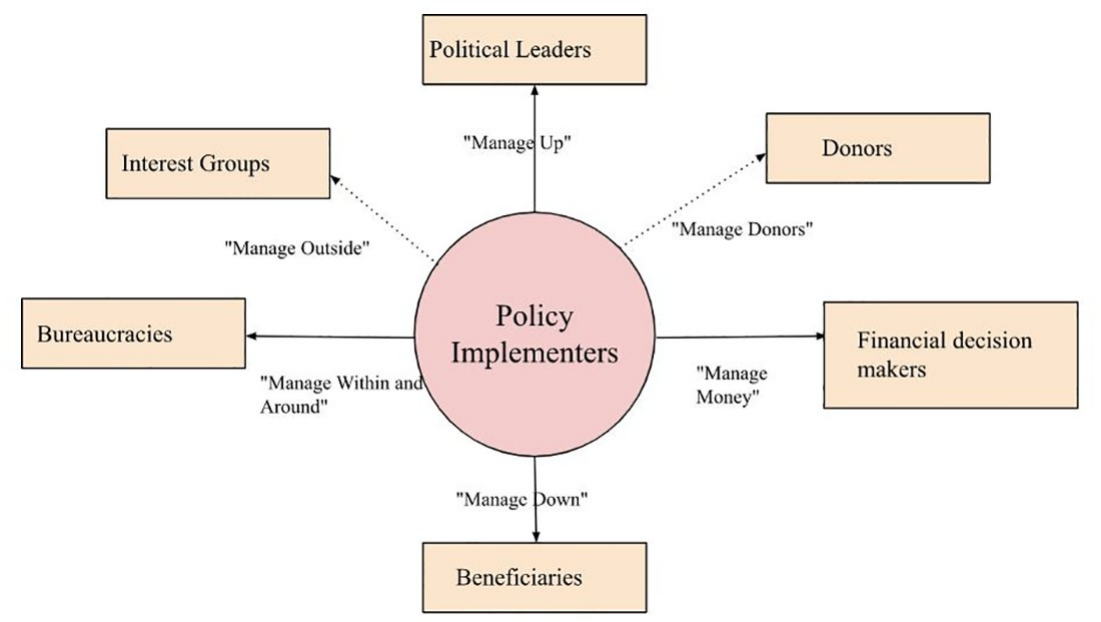
Framework for managing different stakeholders in healthcare policy.

Another important issue was the communication lags and miscommunication between the government and the public. Surprisingly, some of the comments from the patient group complained that patients were not included in the consultation sessions and that this led to less effective communication between stakeholders and policymakers. Referring to Mendelow’s stakeholder mapping in Fig 2, media outlets demonstrated medium interest and low power in the policymaking process because of their limited news coverage, which was believed to have contributed to communication lags. Researchers argued that it was crucial for the government to utilise media outlets such as social media that could contribute to effective healthcare policy communication, as occurred with the COVID-19 vaccine [41]. This could be applied to the context of public health policymaking, in which the context of the policy is relatively complex but requires the understanding of the public. It allows the government to gather support from the public, which then smoothes the progress of policymaking.

Because most of the comments from the UPC have included a range of potential adverse impacts, it has been argued that there is a”collective action dilemma” with an overrepresentation of interest groups, mainly industry representatives, in the public consultation. Although it is important to involve stakeholders who have enough capacity to participate in developing public policy, Riege and Lindsay stated that policymakers should have recognised that this might bear a political risk as a significant portion of the electorate might be diverted easily by their opponents [37]. In keeping with communication and knowledge transfer effectiveness, the UPC comments have shown unresolved issues, such as a need for more transparency and a clearer data source for the CBA process. This was validated by Ashraf and Ong, whose study demonstrated that the policy guidelines had been vaguely expressed and lacked the support of clear evidence [13]. Therefore, transparency and inclusion are necessary to reshape public trust by focusing on the needs of all stakeholders (i.e., general public) [42].

The study illustrates the complex relationship among various stakeholders. When discussing the different perspectives of the socioeconomic, government, and industrial sectors, the results suggested that the ERP could result in overlapping effects among stakeholders. For instance, key informants were concerned that the ERP policy might reduce the profit margins of pharmaceutical companies, which could further lead to a decrease in the research and development of innovative drugs (i.e., industrial factor). The decrease in innovative drugs could hinder the accessibility of medicines for the general public (i.e., socioeconomic factor). This requires adequate management from different stakeholders on health policies, as demonstrated by Campos and Reich [43] (Fig 2). The findings from the study suggested different approaches to the implementation of health policy. It is important for policymakers to identify strategies for implementation and re-examine whether there is a need to create incentives for the interest groups to support the implementation of policy, known as “managing outside” (upper-left arrow in Fig 2). Campos and Reich also suggested that communicating with beneficiaries of the policy regularly is fundamental in policy implementation, which refers to “managing down” (bottom arrow in Fig 2), where this approach could be further explored in the Malaysian context [43].

Ashraf and Ong indicated that the ERP has great potential to succeed as there is a strong political intention from both the incumbent and opposition parties and substantial support from the public and patient groups [13]. Aside from understanding the chance of success, it is crucial to ensure that Malaysia has fulfilled the key criteria to introduce the ERP. Gill et al. have listed 14 criteria as the best practice framework for examining the feasibility of introducing the ERP [14]. These 14 criteria included: clear objectives aligning with policy goals, a focus on on-patent drugs, making sure that ERP prices do not override health technology assessment decisions, simple and transparent administration of the policy, stakeholder participation, allowing the possibility of appeal, appropriate country selection, consideration of international implications, use of ex-factory prices, use of mean prices, respect of patent status, avoidance of the impact of the exchange rate, and keeping price revisions to a minimum and staying in alignment with negotiation tools.

The preliminary results of this research, show that it is clear that Malaysia’s healthcare policy does not align with the three main criteria (out of 14) [14] listed by Gill et al. This information also explains RQ2 and RQ3 of the study:

1. The policy does not have clear objectives that are aligned with policy goals (i.e., stakeholders noted that the proposed ERP was vague, as discussed earlier in this section);
2. The policy needs to be administratively simple and transparent (i.e., stakeholders were concerned about the clarity of the data and methodology used in the CBA analysis);
3. The policy needs better stakeholder participation (i.e., a lack of stakeholder participation was outlined in the introduction).

To sum up, overcoming these issues will not only increase the chances of success but will also improve the effectiveness of the ERP implementation.

Regarding the current Malaysian healthcare policy recommendations (linked to the RQ3 of the study), the key informants in the UPC comments emphasised the need for healthcare reform. Some healthcare professionals argued that the current healthcare system requires a series of reforms, as most of them were not convinced by both the CBA1.0 and CBA2.0 reports. However, these comments indicated a window of opportunity for policymakers to acknowledge the current weaknesses of the healthcare system and identify ways of improving, such as reviewing the patent system, and having a clearer dispensing separation system, as well as needing to introduce universal healthcare coverage. Interestingly, dispensing separation was one of the main concerns of stakeholders. A clear boundary between physicians and pharmacists will significantly reduce medicine prices as it will prevent overprescribing by physicians [44]. A study by Mubarak et al. pointed out the issue of overprescribing and inappropriate prescribing among private physicians as Malaysia does not have dispensing rights separation. This has become one of the barriers to inter-professional collaboration for improving public health outcomes [44]. Also, physicians may have higher financial incentives to dispense expensive medications, resulting in an increase in drug expenditures [45]. It has been shown that the price of generic medication is higher in countries in which physicians retained prescribing rights compared with countries that gave pharmacists the right to dispense medicines [46]. This example is a good reason for policymakers to re-examine the policies for healthcare reform to ensure the feasibility of implementation and provide a chance to discover other policy changes that would lead to better options for controlling domestic medicine prices, such as dispensing rights separation.

It is important to note that some of the concerns mentioned in the UPC comments were affected by the reports in CBA 1.0 and CBA 2.0; it is unlikely for the study to capture the actual perceptions of the community (general public). Although the UPC comments included the main stakeholders’ views on pharmaceutical pricing regulations, most of the UPC comments were biased towards a negative perspective of the implementation of the ERP. Therefore, it is necessary to include more diverse data sources to explore views that the UPC comments could not capture.

We acknowledge that more stakeholders should be included to capture the whole picture of all potential impacts and challenges of implementing an ERP policy. However, this remains a limitation of the study because not all stakeholders are reachable as listed in the stakeholder mapping. The research only explains some of the perceptions of only a few major stakeholders and thus may not generalise the results to a larger population. Future research can cover more stakeholders’ perspectives on ERP policy. To address such limitations, interview data will need to be used to supplement the missing part of the UPC comments in the future study.

## Conclusion

It has always been challenging for the government to improve the efficiency of the healthcare system and enhance the accessibility of high-quality affordable healthcare. Hence, policymakers must better understand the stakeholders involved and the potential impact of ERP implementation on all stakeholders. Evidence from this study has mapped out the potential influence of the diverse stakeholders in the socioeconomic, industrial, and government sectors. Throughout the classification of different stakeholders’ views and recommendations, the implications of these findings provide a direction for policymakers to review current practices of the Malaysian healthcare system and contribute to the existing literature for other countries that experienced similar issues. The study also concludes that stakeholder engagement is the major determinant of decision-making for pharmaceutical policies. More stakeholder involvement is required in the upcoming research plan to provide more comprehensive insights into the implementation of ERP.

## Supporting information

S1 Appendix Stakeholder mapping. (PDF)
