# Supplementary material for: ”What’s up with price controls?” Stakeholders’ views on the regulation of pharmaceutical pricing in Malaysia
Source: PLoS One. 2023 Dec 7;18(12):e0291031. doi: 10.1371/journal.pone.0291031 (PMC10703332; doi:10.1371/journal.pone.0291031)
Supplement: S1 Appendix — (PDF) [file pone.0291031.s001.pdf]

Appendix 1: Stakeholder Mapping

Inclusion Criteria:

1. Possible stakeholders that involve and participate in the pharmaceutical pricing control mechanism;
2. Stakeholders and individuals involved in the evaluation process (e.g., CBA 1.0 & 2.0);
3. Key players and informants of the healthcare system.

High Power -

(1) Has the absolute authority to influence the policy-making process

(2) Key stakeholders that have been consulted **>= 5 times** in government consultations **only**.

Or

(3) Consulted **>=5 times** by the government and involved in **one** of the CBA (1.0 and 2.0) analyses.

or

(4) Consulted **at least once** by the government and involved in **both** the CBA (1.0 and 2.0) analyses.

Medium Power –

(1) Has the relative authority to influence the policy-making process

(2) Key stakeholders that have been consulted **a few times (2-4 times)** in government consultations **only**.

or

(3) Consulted **1-4 times** by the government and involved in **one** of the CBA (1.0 and 2.0) analyses.

or

(4) Never been consulted but involved in **one of** the CBA (1.0 and 2.0) analyses.

Low Power -

(1) Has less or no authority (passively affected) to influence the policy-making process

(2) Consulted **once** in government consultations **only** and **did not** involve in the CBA analyses.

Determinants of interest level: (1) How much are the stakeholders aware of the impact of the policy?

(2) How interested the stakeholder group (their expectations) in the policy (Scholes & Johnson, 2002)

High interest - (1) The policy impacted the stakeholder the most (with its interest).

Or

(2) Showing high interest in the policy outcomes with comments made publicly.

Medium interest- The policy moderately impacted (indirectly affecting) the interests of the stakeholder.

Low interest- The policy itself does not impact the stakeholder much.

Table 1: Mendelow's Stakeholder Mapping.

Stakeholder	***Governance level***	***Key interest***	***Power influence***	***Interest***	***Description***
Public	National	Price of medicines, affordability.	Low	Low	1.General public/Malaysians<br><br>2.The knowledge of the price controls mechanism is not well-broadcasted to the public.
Patients	National	Price of medicines, affordability.	Low	High	1. They are vulnerable groups that are likely to affect by their medical expenses [1].<br><br>2. Consulted in the second analysis, arguably the number of representatives does not enough in the second CBA (2 persons).
Academicians	National	Advocate for general public/professional contributions.	Medium	High	1. Researchers/scholars who conducted research on healthcare policy.

					2. Commentaries have been published in local media as an expression of their standpoint.<br><br>3. Academicians (3 persons) were invited as a consultancy team for the public consultation on medicine controls policy for the second CBA (CBA 2.0).
Pharmacists	Regional	Profitability of their businesses.	Medium	High	1. One of the main medicine suppliers.<br><br>2. Community pharmacists have been consulted 2 times respectively during Jan- Nov 2020 with regard to pharmaceutical pricing regulation policy.
General Practitioners / Physicians	Regional	(1) Profitability of healthcare services;<br>(2) Operational cost of clinics.	Medium	High	1. One of the "key players" in the industry who prescribes medicine for consumers. Their business relies on selling medicines (consultation fees have been regulated by the government.)<br><br>2. Only consulted **once** during the consultation period (first CBA, Jan-Nov 2020).

Multinational Pharmaceutical Companies (e.g: PhAMA)	International	(1) Profitability of their businesses.<br>(2) Expected returns from research and development of new drugs.	High	High	1. Pharmaceutical companies are directly affected by the price control mechanism.<br>2. Make decisions on R&D on life-saving drugs.<br>3. Consulted **10 times** on the policy-making process (for both CBA analyses).
A.P.I Suppliers (e.g: MOPI)	National	(1) Profitability of their businesses.	Medium	High	1. Generic suppliers of Malaysia which **opposed** to originator medicines.<br>2. Consulted **twice** during the consultation period (first CBA, Jan-Nov 2020), involved in the second CBA (CBA2.0) analysis only.
Public Health Facilities (e.g: Public hospitals)	Regional	(1) Quality of healthcare services.<br>(2) Allocation of resources from the government.	Low	Medium	1. Played a **major role** in the distribution of medicines.<br>2. Price controls might affect the decision on medicine procurement, and the allocation of funds from MoH.<br>3. Minimal involvement in the decision-making role of drug price control policy.

Private Health Facilities	Regional	(1) Quality of healthcare services.<br>(2) Profitability of hospital.	Medium	High	1. One of the largest medicines and healthcare "retailers".<br><br>2. Institution(s) that hire large numbers of healthcare workers.<br><br>3. Consulted **2 times** during the consultation period (first CBA, Jan-Nov 2020), and also involved in the second CBA (CBA2.0) analysis only.
Insurance Companies	National	Financial returns from providing insurance coverage.	Medium	High	1. Health insurance providers that cover medical fees (Malaysia do not have universal health care coverage).<br><br>2. Consulted **3 times** during the consultation period, **twice** in the first CBA (Jan-Nov 2020) and **once** in the second consultant period (CBA 2.0) respectively.
Banks/Financial Institutions	National	Returns on investments.	Low	Medium	1. Investor of pharmaceutical companies/healthcare institutions (e.g.: Maybank- KPJ healthcare).<br><br>2. Does not involve in the policy-making process.
Tender Agents	National	Profitability of the business.	Low	Medium	1. An intermediary that brings an agreement between healthcare facilities

					(hospitals) and pharmaceutical companies.<br><br>2. "Despite earning 2-3% in commissions for submitting tender documents on behalf of their suppliers, their exposure is limited and minimal." [2]
Media	National	Spreading information	Low	Medium	1. Only a few media outlets (printed and online publications) cover the policy. (Malaysiakini, CodeBlue, The Malaysian Insight, The Malay Mail)<br><br>2. Act as a broadcaster of government.
Think Tanks	National	Public policy advocacy/ professional engagement.	Low	High	1. Third-party research institutions pointed out the pros and cons, and possible improvements of the policy.<br><br>2.Has a certain level of influence on the policymaking process. However, does not involve in the policy decision process/ government meetings.
Health Ministries	National	Health and well-being of Malaysians.	High	High	1. The main provider of healthcare services in the country, and a key player in policy implementation.
Economic Planning Institutions	National	Government revenue and expenditure (allocation of national funds).<br><br>Economic development of the nation.	High	Medium	1. The main body for economic planning.<br><br>2. Consulted **once** during the consultation period (first CBA, Jan-Nov 2020). However, involved in the second CBA (CBA2.0) analysis.

Ministry of Domestic Trade and Consumer Affairs (KPDNHEP)	National	Consumer welfare/collaboration with MoH on the implementation of the policy.	Medium	Medium	1. Responsible for policy implementation (price controls).<br><br>2. Held **5 meetings/consultations** with the government during the first CBA analysis (Jan-Nov 2020).
Ministry of International Trade and Industry (MITI)	National	Investment, the productivity of small and medium enterprise/ Collaboration with MoH on the implementation of the policy.	High	Medium	1. To ensure fair trade and competition in the healthcare industry.<br><br>2. Held **4 meetings/consultations** with the government during the first CBA analysis (Jan-Nov 2020); Involved in the evaluation of the policy (CBA 2.0).
Intellectual Property Corporation of Malaysia (MyIPO)	National	Intellectual property (IP) rights of involved institutions.	High	Medium	1. The main body for IP rights/ commercialises research (innovative drugs).<br><br>2. Involved in the evaluation of the policy.<br><br>**Note:** Research argued that under the current patent system, imposing a pricing control policy would further reduce the availability of innovator drugs [3,4], suggesting the number of patent applications might *decrease* with a price control policy.

Social Security Providers/Institutions	National	Public welfare.	High	Medium	1. Provides social security protection for employees in the private sector.<br>2. Responsible for the adjustments to the social security scheme.<br><br>**Note:** Malaysians can withdraw a certain amount of money from EPF for healthcare expenses [5].

References

1. Beran D, Pedersen HB, Robertson J. Noncommunicable diseases, access to essential medicines and universal health coverage. Global health action. 2019 Jan 1;12(1):1670014.

2. Galen Centre for Health and Social Policy. Policy Brief: Drug Price Controls In Malaysia: Implications and Considerations [Internet].2019 June [cited 2022 Aug 15]. Available from: https://codeblue.galencentre.org/2019/05/02/minister-dzulkeflys-achievements-and-what-they-really-mean、

3. Lanjouw, J.O. Patents, Price Controls, and Access to New Drugs: How Policy Affects Global Market Entry. NBER Working Paper Series. 2005;(11321). Available from: https://doi.org/10.3386/w11321

4. Kakkar AK. Pharmaceutical price regulation and its impact on drug innovation: mitigating the trade-offs. Expert Opinion on Therapeutic Patents. 2021 Mar 4;31(3):189-92.

5. Hassali MA, Tan CS, Wong ZY, Saleem F, Alrasheedy AA. Pharmaceutical pricing in Malaysia. Pharmaceutical prices in the 21st century. 2015:171-88.
